# Modern day superheroes

**DOI:** 10.36834/cmej.70721

**Published:** 2020-09-23

**Authors:** Andrew Seal

**Affiliations:** 1 University of British Columbia, British Columbia, Canada

**Figure:**
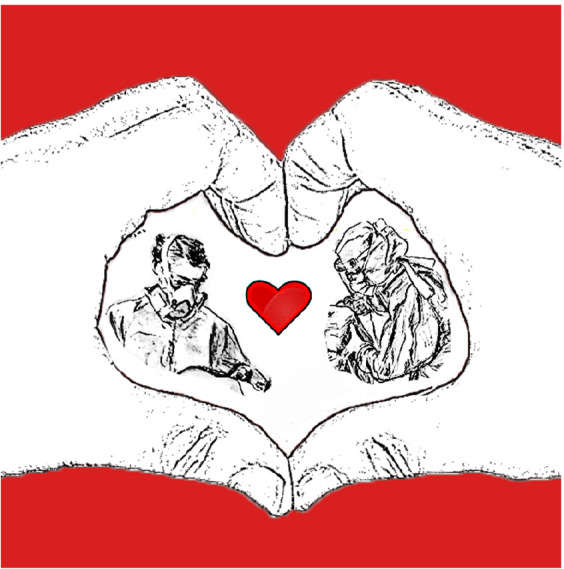


During National Nurses Week 2020 I posted this image to my blog *thechangingpalette* to say thank you to a lifetime of nursing colleagues and dear friends and to thank all those working for all of us day and night on the frontline during this COVID-19 pandemic. They are all true modern day superheroes.

